# CPL photoscopy: circularly polarized luminescence detected by chromaticity differences†

**DOI:** 10.1039/d5sc03949e

**Published:** 2025-07-14

**Authors:** Matteo Cei, Francesco Zinna

**Affiliations:** a Dipartimento di Chimica e Chimica Industriale, Università di Pisa Via Moruzzi 13 Pisa 56124 Italy francesco.zinna@unipi.it

## Abstract

Circularly polarized luminescence (CPL) is attracting growing interest for a wide range of applications, spanning from materials with advanced optical properties to innovative imaging techniques. The traditional approach to CPL consists in measuring the emission intensity difference between the left and right polarizations of light and usually requires spectral separation through color filters or a monochromator. In this work, we show that, under certain circumstances, it is possible to extract CPL information simply from the chromaticity values of a pair of photographs taken under different polarizations. This novel technique requires cheap instrumentation, offers complementary features with respect to the traditional method and could potentially be applied to CPL imaging.

## Abstract 2

Circularly polarized luminescence (CPL) is always determined through differential intensities for left and right polarized emission. Here we show that CPL information encoded in *rgb*-chromaticity can be extracted from pairs of photographs.

## Graphical abstract

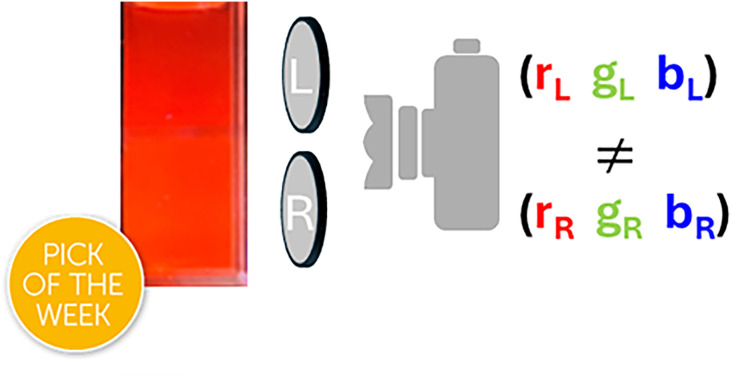

## Introduction

Circularly polarized luminescence (CPL) is unlocking more and more opportunities in photonic and optoelectronic devices and life and materials sciences.^1^ Examples range from circularly polarized (CP) OLEDs for antiglare displays,^2–6^ and CPL inks and tags for security applications,^7–12^ to CPL assays *in vitro*^13–15^ and cell staining.^16^ In recent years, the opportunities for CPL applications have increased thanks to new methods of CPL generation (*e.g.* through electrochemical techniques^17–20^ and 2-photon excitation^9,21–24^), as well as new techniques allowing for CPL (confocal) microscopy or imaging, tracking polarized emission from chiral probes and exploiting the concept of enantioselective differential chiral contrast.^16,23,25,26^ Moreover, significant efforts have been devoted to developing cheaper alternatives for CPL detection, available beyond specialized laboratories, and in principle even to the broader public. Commonly, CPL is measured by commercial or homemade spectropolarimeters where the polarization is analyzed through photoelastic modulators by means of lock-in detection.^2,27–29^ Despite their high accuracy and sensitivity this type of instruments are bulky and expensive (in the order of 100 k€). On the other hand, recently much cheaper alternative setups have been proposed.^30^ Examples range from ultracheap detectors,^31^ CP light-sensitive transistors and photovoltaic elements,^32^ setups separating polarizations using beamsplitters^33^ and CCD cameras,^34^ to handheld devices with built-in grid polarizer arrays, to be used for CPL imaging in materials and life sciences.^35^ These techniques are intended to help circularly polarized technologies transition towards widespread and general use.

A common feature of all these methods, as well as the more standard ones, is that the detection always relies on recording a difference in intensity of two light beams encoding respectively left and right circular polarization (*I*_L_ and *I*_R_). CPL is then quantified by metrics such as the dissymmetry factor:1
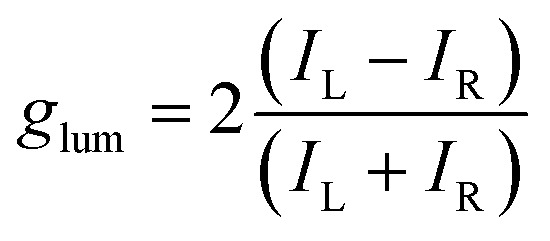
and circularly polarized brightness (*B*_CPL_),^36^ which takes into account the extinction coefficient (*ε*_exc_) and the quantum yield (*φ*) of the emitter:2
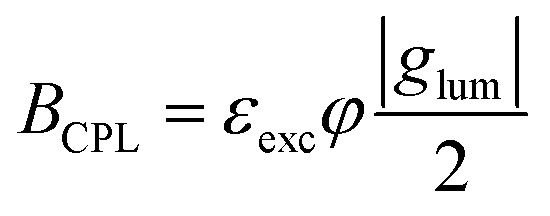


For the purposes of enantioselective CPL imaging, large *g*_lum_ and *B*_CPL_ are desirable to maximize contrast and total signal respectively.^35^

In most cases, a spectral separation of the emitted light needs to be achieved, through monochromators or at least bandpass or color filters, prior to the detection.^37,38^ This is especially necessary when the polarized signal is accompanied by non-polarized bands (such as background fluorescence^39^), or when the luminescent species has oppositely polarized bands, as in the case of chiral lanthanide complexes. These issues may be mitigated through time-gated detection of CPL,^26,33,35^ to eliminate short-lived unpolarized background signals and, where possible, by designing CPL-active species with single sign CPL.^40^

In the following, we propose a conceptually different method for CPL detection, which does not require the selection of single bands. In contrast to all the methods experimented with so far, we aim at detecting CPL signals encoded in color (or more precisely chromaticity) differences, rather than directly measuring differential intensities between *I*_L_ and *I*_R_.

Commercial digital cameras provide color images thanks to a built-in color filter array. Therefore, in general a digital photo of any (non-polarized) absorbing or fluorescent sample naturally encodes spectral features, which may be extracted by simple mathematical treatments. Despite being a low cost and fast technique, the camera's sensitivity allows for fast and cheap screening of color evolution in a sample or batch-to-batch color differences.^41–43^ These colorimetric experiments have sometimes been termed *photoscopy* in the literature, especially when performed using color cameras.^44^

**Fig. 1:**
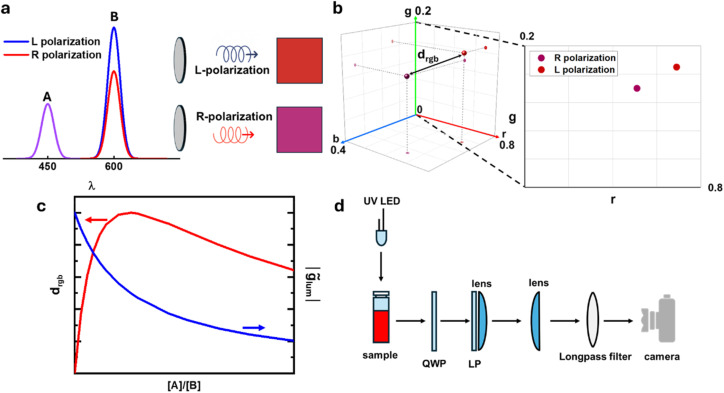
(a) Illustration of the base principle of CPL photoscopy, with resulting colors under L and R circular polarization. (b) *rgb* coordinates for the two polarizations on a 3D and a 2D plot. (c) Calculated trend of *d*_rgb_ and *g̃*_lum_ as a function of the [*A*]/[*B*] emission intensity ratio. (d) Schematic of the experimental setup for the acquisition of the photographs.

Can this concept be extended and applied to detect CPL by means of simple photography? Here we show that it is possible to recover CPL information through a commercial digital color camera when the sample shows at least a polarized band and at least a second band with a different polarization. The second band (i) may have the same sign but a different polarization degree, or (ii) opposite sign polarization or (iii) may be completely non-polarized. The principle lies in the fact that under these circumstances two pictures of the whole emitted wavelengths, taken under different polarizations (*i.e.* under a left/right circular polarizer), will show a difference in chromaticity (see Fig. 1a). Such bands may come from the same fluorophore or from different fluorophores. Particularly relevant may be the case of a CPL-active probe and ambient/background non-polarized fluorescence.

Our method consists in simply taking a pair of snapshots of the emitting sample behind a circular polarizer, without a monochromator or color filters, and comparing the chromaticity of the pictures obtained under left and right polarization. A schematic of the experimental setup for the acquisition of the photographs is depicted in Fig. 1d. The difference in chromaticity encodes the polarization of the sample. The concept is illustrated in Fig. 1a in the case of a non-polarized band at shorter wavelength (*A*, with *I*_L_ = *I*_R_) and a polarized one at longer wavelength (*B*, *I*_L_ > *I*_R_). The total emission color will appear different if observed through a left-polarizing (red-like) or a right polarizing filter (magenta), as the ratio between the red and blue components of the overall spectrum depends on the polarization under which the sample is observed. This difference in color can be precisely quantified, as each digital photo consists mathematically of 3 overlayed pixel matrices encoding intensities for red (*R*), green (*G*) and blue (*B*). *RGB* values found in each pixel of the image define the emerging color of that pixel. Chromaticity coordinates (*r*, *g*, *b*) represent color without brightness information and can be extracted from *RGB* values by a simple normalization:^45^3
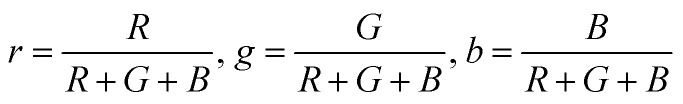


Each *rgb* point can be plotted in a 3-dimensional graph or in a 2-dimensional one featuring *r* and *g* on the horizontal and vertical axes respectively (Fig. 1b). Note that the *b* component is always determined, since *r* + *g* + *b* = 1. In the example in Fig. 1, *rgb* chromaticity coordinates observed under a left (*r*_L_, *g*_L_, *b*_L_) and a right polarizing filter (*r*_R_, *g*_R_, *b*_R_) will correspond to two distinct points. The polarization information is thus encoded into the normalized Euclidean distance (*d*_rgb_) between such pairs of points as:4
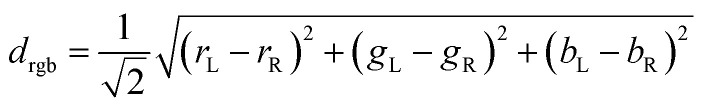
In the presence of at least two bands meeting one of the above-mentioned conditions, in principle *d*_rgb_ ≠ 0 for the chromaticity of the photographs taken under left and right polarization. Note that *d*_rgb_ (0 ≤ *d*_rgb_ ≤ 1) is a metric informing about the extent of chromaticity contrast, due to the CPL activity of the sample, available for detection.

Another way to visualize these data may consist in plotting Δ*r* and Δ*g*, defined as:

5 Δ*r* = *r*_L_ − *r*_R_ and Δ*g* = *g*_L_ − *g*_R_

in a bidimensional plot, with Δ*r* and Δ*g* ranging from −1 to 1, in contrast with *d*_rgb_ which is always positive. In this type of plot, the points corresponding to two enantiomers should appear symmetrical with respect to the axis origin, while in the absence of any polarization the point should lie at the axis origin (see Fig. S1 for an example†).

Such method may be referred to as *CPL photoscopy* and, being based on a completely different detection principle, it offers complementary features with respect to the standard CPL spectroscopic techniques based on differential intensities. To appreciate its main advantage, it is instructive to consider what happens to *d*_rgb_ when the intensity of the non-polarized band (*A*, see Fig. 1a) increases with respect to the polarized one (*B*). When *A* = 0 also *d*_rgb_ = 0, as the chromaticity of the single polarized band *B* does not change with the polarization chosen (only absolute intensity does). When *A* ≠ 0, *d*_rgb_ starts increasing by increasing *A* intensity up to a point (maximum contrast), then it decreases again as the non-polarized *A* signal becomes dominant over the polarized *B* one (Fig. 1c). This is in sharp contrast with respect to the trend followed by *g̃*_lum_ calculated over the total spectral range (*i.e.* the *g*_lum_ that is obtained in the absence of color bandpass filters):6
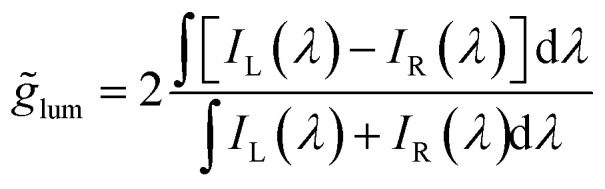
which monotonically decreases upon increasing *A* intensity (Fig. 1c). In a practical situation, relevant to imaging, *A* may be the non-polarized fluorescence from the background and *B* the polarized signal from a CPL-reporter molecule. With the intensity-based standard methods, *A* would interfere with CPL detection, decreasing the CPL-imaging contrast (quantified by *g̃*_lum_) unless attenuated with time-gate methods or bandpass filters, while in CPL photoscopy, up to a certain intensity ratio, *A* enhances the signal (quantified by *d*_rgb_). A full tutorial on how to extract and treat the photographic raw data is given in the ESI.†

## Results and discussion

**Fig. 2:**
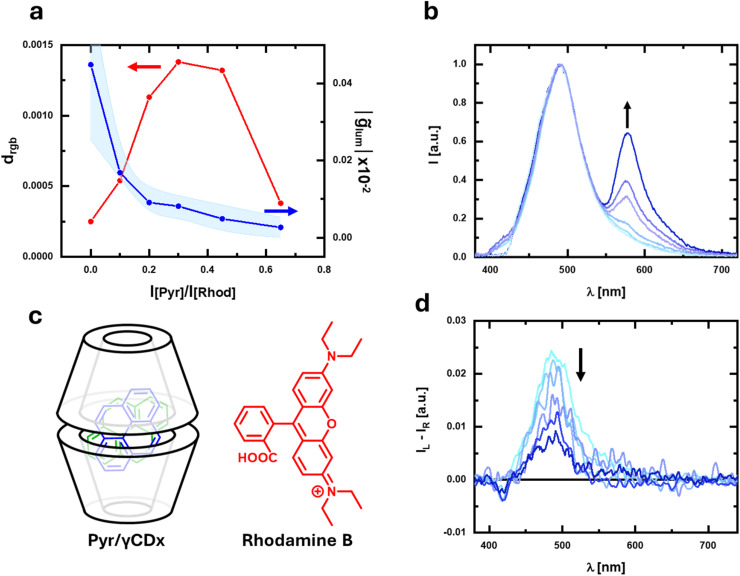
(a) Plot of *d*_rgb_ (red) and *g̃*_lum_ (blue) for a solution of pyrene/γ-CDx containing increasing amounts of rhodamine B. (b) Emission and (d) corresponding CPL spectra of the solution of pyrene/γ-CDx containing increasing amounts of rhodamine B; the CPL spectra are normalized by the integral of the corresponding total emission spectrum. (c) Pyrene/γ-CDx adduct and rhodamine B.

To prove this concept in practice, we started by investigating the case of an aqueous solution containing pyrene and γ-cyclodextrin (γ-CDx).^46^ This solution is known to give a CPL signal (*λ*_max_ = 485 nm, *g*_lum_ = 2.5 × 10^−2^, Fig. 2d) associated with excimer formation due to the stacking interaction of pyrene molecules within the chiral γ-CDx cavity (Fig. 2c). We took snapshots of the sample under 365 nm excitation from a LED source with a commercial digital camera (Nikon D3500, 12-bit color depth). Pairs of pictures were taken by rotating by 90° a quarter waveplate (QWP) with respect to a fixed linear polarizer (LP) to select the left and right circularly polarized components of the total emission. The QWP axis was aligned at ±45° with respect to the LP axis. As expected, no significant difference in chromaticity is observed in pictures taken through left/right polarizations (*d*_rgb_ ≈ 0, Fig. 2a), since the emission spectrum shows only a single band. On the other hand, *d*_rgb_ quickly increases by adding increasing quantities of rhodamine B, which gives a non-polarized band centered around 575 nm. Note that rhodamine B, unlike pyrene, does not interact with γ-CDx (Fig. 2b, d and S8†). *d*_rgb_ reaches its maximum (1.4 ± 0.4) × 10^−3^ when the [rhodamine B]/[pyrene] emission intensity ratio is ≈0.3. As expected, a further increase of rhodamine B concentration decreases *d*_rgb_ (Fig. 2a). On the other hand, the *g̃*_lum_, calculated for the whole spectral range (380–720 nm) according to eqn (6), is maximum when no rhodamine B is added, and the spectrum shows a single polarized band. As expected, the addition of increasing amounts of optically inactive chromophore causes a monotonic decrease in the *g̃*_lum_ value (Fig. 2a), *i.e.* of the overall dissymmetry factor stemming from the *I*_L_/*I*_R_ intensity difference detected under the same conditions as CPL photoscopy. Note that the *g*_lum_ value of the pyrene excimer band is independent of rhodamine B addition.

This example shows that despite the very small chromaticity differences (*d*_rgb_ ≈ 10^−3^) due to the relatively small polarization of the pyrene excimer band, a commercial camera is able to detect a chromaticity contrast for the two circular polarizations. Under our conditions the minimum detectable *d*_rgb_ was quantified to be ≈4.8 × 10^−4^, estimated as 3 times the standard deviation of *d*_rgb_ values taken from photographs of a non-polarized white LED source.

**Fig. 3:**
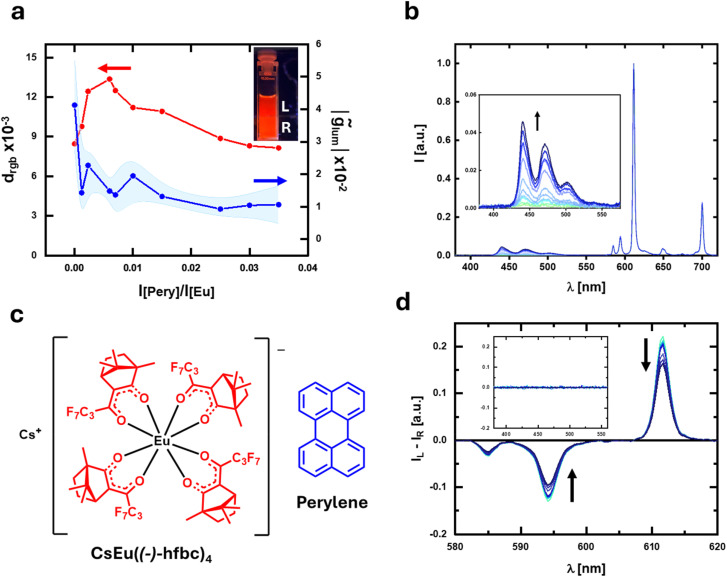
(a) Plot of *d*_rgb_ (red) and *g̃*_lum_ (blue) for a solution of CsEu((−)-hfbc)_4_ containing increasing amounts of perylene. Inset: photo of a CsEu((−)-hfbc)_4_/perylene solution irradiated with a 365 nm source, seen through an L and R circular polarizer (upper and lower part of the photo). (b) Emission and (d) corresponding CPL spectra of the solution of CsEu((−)-hfbc)_4_ containing increasing amounts of perylene; the CPL spectra are normalized by the integral of the corresponding total emission spectrum. (c) CsEu((−)-hfbc)_4_ and perylene.

CPL photoscopy may also prove very useful in the case of chiral emitting lanthanide complexes, such as red-emitting Eu(iii)-based compounds.^47–50^ Thanks to their highly polarized transitions and bright red emission,^51–53^ chiral Eu complexes are often used in chiral electronics, *in vitro* probes^13,14,54,55^ and chiral stains in cells for polarized imaging or microscopy,^16^ and possibly in polarized inks.^7^ Chiral Eu(iii) complexes usually feature several sharp bands with opposite polarization. This is a limitation with the traditional CPL detection method, as it leads to extensive signal cancellation, unless a selected band is observed through a narrow bandpass filter, which in turn decreases the amount of collected photons. On the other hand, such complexes naturally meet the requirements for CPL photoscopy detailed above, even in the absence of bands from additional fluorophores. We show this concept by taking as an example CsEu((−)-hfbc)_4_ (hfbc = heptafluorobutyrylcamphorate, Fig. 3c) in CH_2_Cl_2_ solution under 365 nm excitation. This complex shows several emission bands in the red region (Fig. 3b), with the main contributions being the ^5^D_0_ → ^7^F_1_ (at 595 nm) and ^5^D_0_ → ^7^F_2_ (at 614 nm) transitions. The associated CPL spectrum (Fig. 3d) shows bands with *g*_lum_ of −1.4 and 0.24, respectively. These two bands have opposite polarization but a similar *B*_CPL_ each,^36^ so that the overall *g̃*_lum_, measured in the 380–720 nm spectral range (*i.e.* without selecting a single band), is vanishingly small (0.04).

Taking a pair of photographs under left and right polarization, we could observe a significant difference in chromaticity quantified by a *d*_rgb_ of (8.5 ± 0.4) × 10^−3^. Indeed, the CPL band at 614 nm is positive (*I*_L_ > *I*_R_) while the one at 595 nm has a negative sign (*I*_R_ > *I*_L_). Thus, in the total emission spectrum, the intensity ratio between the 614 and 595 nm bands will be lower under right polarization and higher under left polarization (Fig. S9†), leading to different chromaticities.^56^

**Fig. 4:**
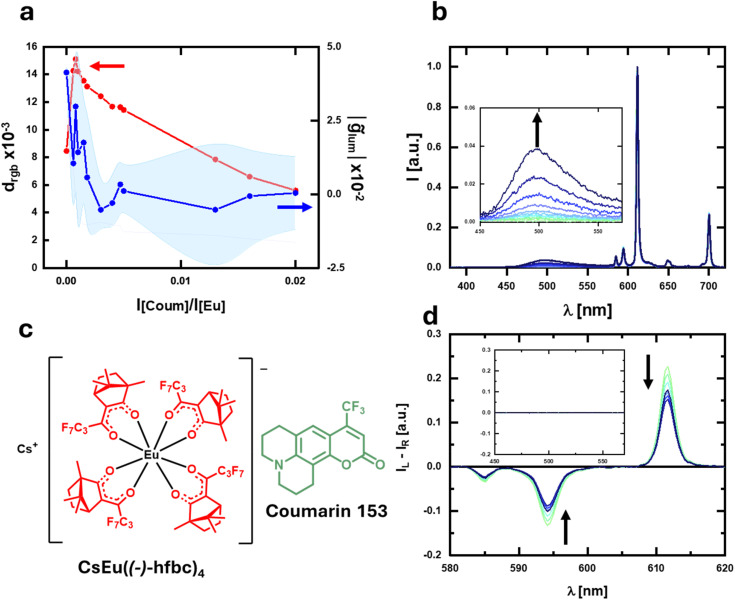
(a) Plot of *d*_rgb_ (red) and *g̃*_lum_ (blue) for a solution of CsEu((−)-hfbc)_4_ containing increasing amounts of coumarin 153. (b) Emission and (d) corresponding CPL spectra of the solution of CsEu((−)-hfbc)_4_ containing increasing amounts of coumarin 153; the CPL spectra are normalized by the integral of the corresponding total emission spectrum. (c) CsEu((−)-hfbc)_4_ and coumarin 153.

CPL active Eu complexes are used as reporters or probes *in vitro* and *in cellulo*, where there may be the interference of background fluorescence. To mimic such a situation, we added again non-polarized light emitting fluorophores in increasing quantities to CsEu((−)-hfbc)_4_. We investigated the system in the presence of blue emitting perylene (*λ*_max_ = 440 nm, Fig. 3c) and green emitting coumarin-153 (*λ*_max_ = 495 nm, Fig. 4c) in dichloromethane solution (Fig. 3b, d, 4b and d). In both cases, *d*_rgb_ showed the expected ascending/descending trend as a function of the optically inactive fluorophore concentration, with a maximum of (1.3 ± 0.6) × 10^−2^ and (1.5 ± 0.1) × 10^−2^ in the case of addition of perylene and coumarin-153, respectively (Fig. 3a and 4a). Again, the *g̃*_lum_ calculated on the whole spectral range (380–720 nm) sharply decreases upon addition of the optically inactive fluorophore, already plateauing for [Pery]/[Eu] (or [Coum]/[Eu]) intensity ratio ≈0.005 (Fig. 3a and 4a).

As a control experiment, we repeated a similar series with an optically inactive complex (Eu(TTA)_3_Phen, TTA = trifluorothenolylacetate) and increasing quantities of perylene. As expected, negligible *d*_rgb_ values (<4 × 10^−4^) were obtained with no significant variation with increasing quantities of perylene (Fig. S10†).

**Fig. 5:**
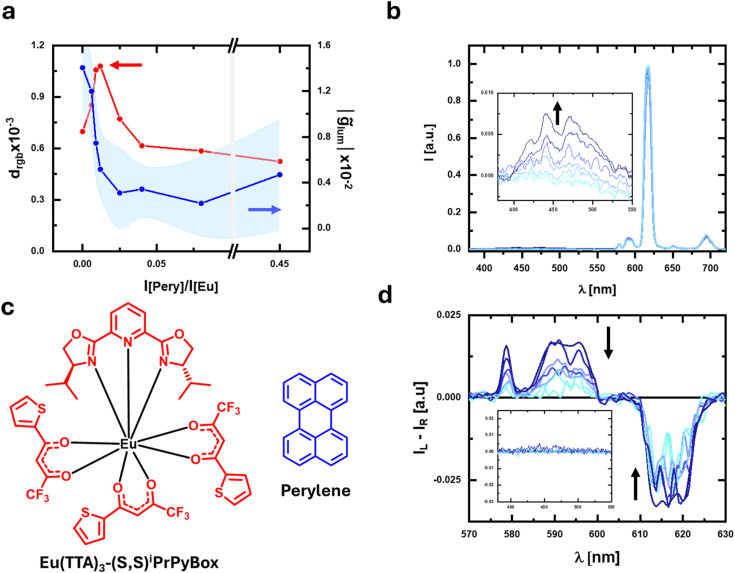
(a) Plot of *d*_rgb_ (red) and *g̃*_lum_ (blue) for a series of PMMA/Eu(TTA)_3_(*S*,*S*)-^i^PrPyBox films containing increasing amounts of perylene. (b) Emission and (d) corresponding CPL spectra of the solution of Eu(TTA)_3_(*S*,*S*)-^i^PrPyBox containing increasing amounts of perylene; the CPL spectra are normalized by the integral of the corresponding total emission spectrum. (c) Eu(TTA)_3_(*S*,*S*)-^i^PrPyBox and perylene.

Finally, to test the general applicability of this method in samples possibly more relevant for life and materials sciences, we investigated the detection performance of CPL photoscopy in solid state samples. We prepared a thin film of polymethylmethacrylate (PMMA) containing 50% of a bright chiral Eu complex, Eu(TTA)_3_(*S*,*S*)-^i^PrPyBox (^i^PrPyBox = isopropylpyridine bisoxazoline, Fig. 5c). We then prepared 8 thin films of Eu(TTA)_3_(*S*,*S*)-^i^PrPyBox in PMMA containing increasing quantities of perylene (from 0% to 2.4%, Fig. S12†). As in the case of CsEu((−)-hfbc)_4_, Eu(TTA)_3_(*S*,*S*)-^i^PrPyBox shows opposite polarization bands, with, in particular, a positive band at 595 nm and a negative one at 614 nm (Fig. 5b and d) with an associated *g*_lum_ of 0.3 and −0.015 respectively. Such opposite bands lead again to a significant cancellation of the overall CPL signal if single bands are not selected (*g̃*_lum_ calculated for the whole spectral range ≈0.014). In the thin film series containing perylene, the maximum *d*_rgb_ was reached for a [perylene]/[Eu(TTA)_3_(*S*,*S*)-^i^PrPyBox] intensity ratio of 0.02, with a value of (1.1 ± 0.76) × 10^−3^ (Fig. 5a).

Is it possible to predict *a priori*, *i.e.* from spectral data, whether a CPL active system will be suitable for CPL photoscopy? To do that, it is necessary to know the camera spectral response for each RGB channel, which is done by determining the pseudo color matching functions (PCMF). See the ESI† for the detailed procedure for determining the PCMFs for a Nikon D3500 camera. Through a convolution of the spectra obtained under left and right polarization with the PCMFs, *rgb* values and *d*_rgb_ are obtained (see the ESI†). Through such a procedure, we were able to reproduce the *d*_rgb_ trends observed in the examples discussed above (Fig. S13–S15†). It is also possible to use this procedure to predict from CPL spectral data whether a CPL-active system or blend can be suitable for CPL photoscopy and which intensity ratio of polarized/unpolarized bands will maximize *d*_rgb_.

Finally, to estimate the potentiality and limitations of CPL photoscopy, by using the camera PCMFS, we calculated the expected *d*_rgb_ for polarized/unpolarized bands as a function of their spectral distance (Δ*λ*) and *g*_lum_ factor of the polarized band (Fig. S17†). Moreover, by knowing the emission spectrum of a certain blend, it is also possible to extract from CPL photoscopy data the *g*_lum_ associated with a single emission band through a calibration procedure. In Fig. S20,† we show an example extracting the *g*_lum_ of the excimer pyrene band in the Pyr/CDx with a rhodamine B mixture.

## Conclusions

In conclusion, we have shown that from a pair of photographs, obtained under L/R circular polarizers, it is possible to extract CPL information, even for systems with an intrinsically low polarization. This can be obtained simply and inexpensively with an entry level color camera. Given the peculiarity of CPL photoscopy, with respect to all the intensity-based methodologies used so far, it offers complementary features which may be advantageous for certain applications. We envision that this technique may be profitably employed in CPL imaging in real samples exploiting the naturally occurring presence of non-polarized background fluorescence, or when using CPL-probes characterized by opposite sign CPL bands, as in the case of lanthanide complexes. Other possible applications are in the field of CPL security inks and tags, with blends containing polarized and non-polarized emitters, whose ratio (and/or optical purity) may change as a function of external stimuli.

Moreover, CPL photoscopy does not require an accurate estimation of *I*_L_/*I*_R_, with chromaticity being a relative quantity; thus it allows CPL information to be decoded even when the emission is not constant in time, such as in some instances of CP electroluminescence or electrochemiluminescence.

## Author contributions

MC: data curation, formal analysis, investigation, writing the original draft/review & editing; FZ: conceptualization, funding acquisition, methodology, project administration, supervision, writing the original draft/review & editing.

## Conflicts of interest

There are no conflicts to declare.

## Supplementary Material

SC-016-D5SC03949E-s001

## Data Availability

The data supporting this article have been included as part of the ESI.†
