# Supplementary material for: CPL photoscopy: circularly polarized luminescence detected by chromaticity differences
Source: Chem Sci. 2025 Jul 14;16(34):15347–54. doi: 10.1039/d5sc03949e (PMC12326326; doi:10.1039/d5sc03949e)
Supplement: SC-016-D5SC03949E-s001 [file SC-016-D5SC03949E-s001.pdf]

Supplementary Information (SI) for Chemical Science.
This journal is © The Royal Society of Chemistry 2025

CPL Photoscopy: Circularly Polarized Luminescence Detected by Chromaticity Differences

Matteo Cei, Francesco Zinna*

Dipartimento di Chimica e Chimica Industriale, Università di Pisa, Via Moruzzi 13, Pisa 56124, Italy.
Email: francesco.zinna@unipi.it

Synthesis of europium complexes

The synthesis of $Eu(TTA)_3$, $Eu(TTA)_3Phen$, and $Eu(TTA)_3$-(*S,S*)^{i}Pr-PyBox was carried out following the literature procedure by Willis et al.[1] The synthesis of the *(−)*-hfbc ligand was carried out according to the procedure by Müller et al.[2] The synthesis of CsEu(*(−)*-hfbc)$_4$ followed the procedure by Zinna et al.[3] The synthesis of $Tb(PMIP)_3$ followed the procedure by Chen et al.[4]

Preparation of pyrene/γ-cyclodextrin solution

0.5 mg of pyrene and 39 mg of γ-cyclodextrin were added to 4 mL of H_2O and the mixture was stirred vigorously. After 12 h the solution was filtered on Hydrophilic Fluoropore (PTFE) 0.45 µm Membrane syringe filter. The obtained solution emitted bright blue light under irradiation from a 365 nm source.

Preparation of rhodamine B/γ-cyclodextrin solution

0.5 mg of rhodamine B and 39 mg of γ-cyclodextrin were added to 4 mL of H_2O and the mixture was stirred vigorously for 12 h.

Preparation of perylene/CsEu(*(–)*-hfbc)$_4$ and coumarin 153/CsEu((-)-hfbc)$_4$ solutions

10 mg of CsEu(*(–)*-hfbc)$_4$ were dissolved in 2 mL of dichloromethane, then 0.5 mL of the solution were diluted with 1.5 mL of dichloromethane to obtain a $2\cdot 10^{-5}$ M solution. To this solution, increasing amounts of a $3\cdot 10^{-6}$ M perylene solution or of a $2\cdot 10^{-5}$ M coumarin 153 solution were added.

Preparation of $Eu(TTA)_3$(*S,S*)-^{i}Pr-PyBox/perylene films

50 mg of PMMA and 25 mg of $Eu(TTA)_3$(*S,S*)-^{i}PrPyBox were added to 1 mL of toluene. The mixture was heated under stirring until the complete dissolution of the solids. To this solution increasing amounts of a $4\cdot 10^{-5}$ M perylene solution were added. After each addition an aliquot of 150 µL was used to prepare a film. To prepare the films, 150 µL of the prepared solution were spin-coated onto a clean glass slide (5 mm x 5 mm, spinning time: 45 seconds, 3000 rpm).

Δg vs Δr graph

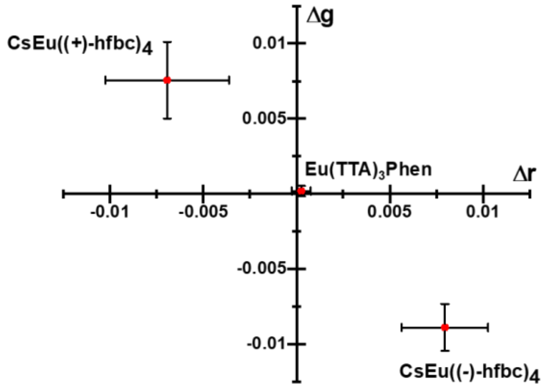

Figure S1. Δr/Δg plot for the two enantiomers of CsEu(hfbc)$_4$ and $Eu(TTA)_3Phen$. The two enantiomers are symmetrical with respect to the origin, while the Δr and Δg for the optically inactive $Eu(TTA)_3Phen$ are ≈ 0.

Photoscopy and spectroscopy: setup

The schematic of the setup used for the photoscopy and spectroscopy measurements is shown in Figure S2. A 365 nm LED source was employed to irradiate the sample. The emitted light from the sample passes through a quarter-wave plate and a linear polarizer. A lens focuses the radiation onto a second lens which collimates the light into the camera objective or into an optical fibre connected to the spectrometer. A longpass filter (cut-on 400 nm) is included in the optical path to eliminate shorter wavelengths from the LED source.

The circular polarization was selected by 90° rotations of a Thorlabs achromatic 400-800 nm QWP places in front to a fixed LP, with relative axes at ±45°.

The effects of the linear birefringence of the Camera CCD on the $d_{rgb}$was assessed by taking snapshots of a non-polarized white LED source for different position of the LP. The d_{rgb} measured for orthogonal orientation of the LP was ≈$3.8\cdot10^{-4}$, i.e. below our detection limit ($4.8\cdot10^{-4}$). Note that this issue in any case may be solved by rotating the QWP while keeping the LP fixed.

Emission spectra were recorded with an Optosky ATP2000 CCD spectrometer. All photographs were taken with a Nikon D3500 camera (12-bit color depth) deprived of the objective, using an ISO value of 100-200, a shutter speed between 0.1 s and 0.025 s, the white correction was set on ‘auto’. All the photographs were saved in a RAW format (.NEF). The pictures were processed in ImageJ 1.54k with plugin DC Raw Reader 1.16.0.1. It is important to note that rgb values and related quantities are specific to this camera model and may differ slightly if other models are used.

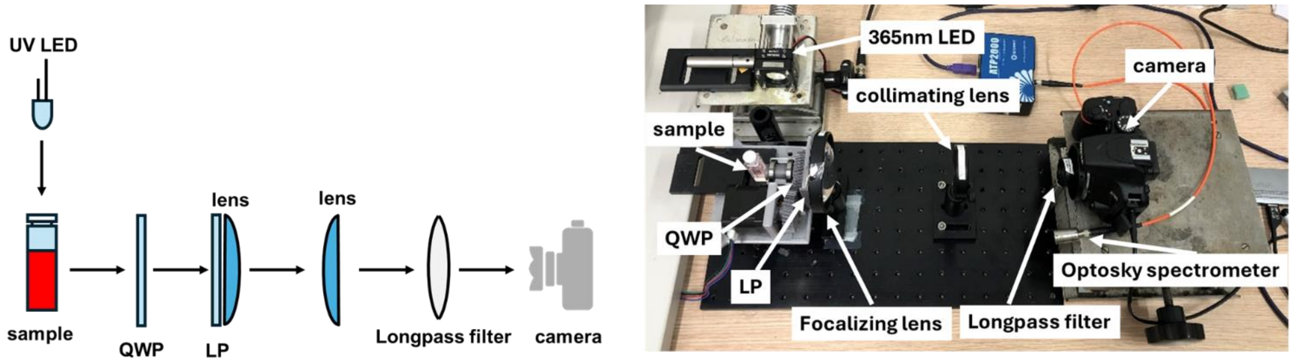

Figure S2. Schematic (left) and picture (right) of the experimental setup used to register the emission spectra and to take photographs of the samples.

Table S1. Estimation of the price of the components for the acquisition of the photographs

Component	Model/characteristics	Price
**365 nm LED**	Thorlabs, M365D1	~100 €
**QWP**	Thorlabs, AQWP05M-600	800 €
**Linear Polarizer**	Thorlabs, LPVISE2X2	20 €
**Lenses**	d=80, f=100 and d=45, f=100 mm	~50 €
**Longpass filter**	Edmund, #47-614	100 €
**Rotational mount+step motor**	Home-made (3D-printed)	~50 €
**Camera**	Nikon D3500	400 €
**TOTAL**		**~1500 €**

Photoscopy: data processing

Notice: it is essential to take RAW pictures, other formats (jpeg, png, etc.) are obtained through non-linear transformations of raw data and may not be suitable for a sufficiently accurate analysis.

1) <u>Preliminary check:</u> for each photograph the value of each RGB coordinate should not exceed the highlight linearity limit of the camera, which can be found in the ‘Log’ window (Plugins>Input-Output>DCRaw Identify), as shown in Figure S3.

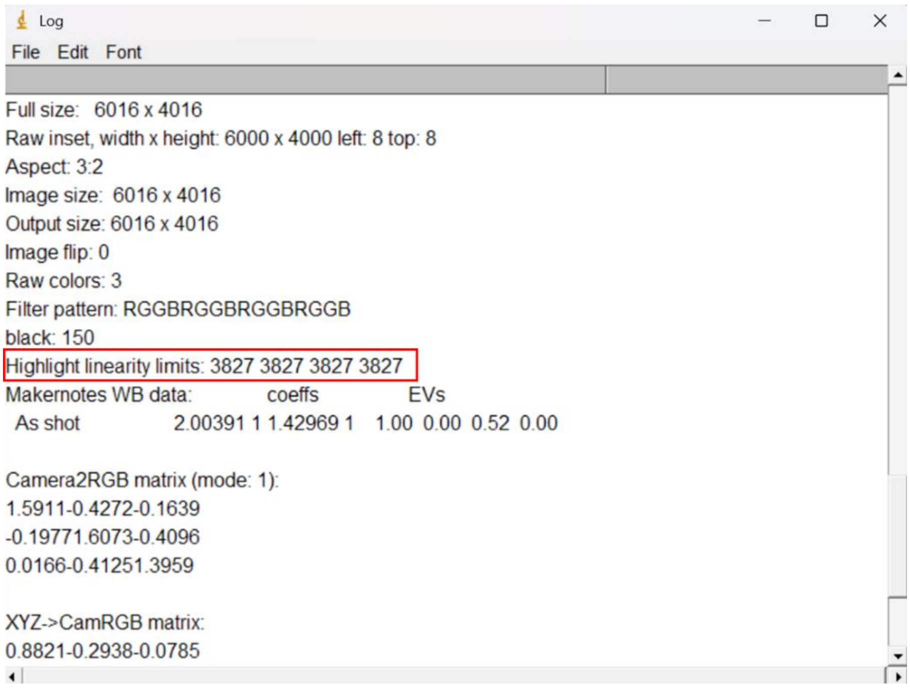

Figure S3. Log window for the Nikon D3500 camera. Highlight linearity limits values are highlighted in red.

2) Extraction of RGB values from photos: each picture was opened in ImageJ by using DC Raw Reader plugin (Plugins>Input-Output>DCRaw Reader). The used settings are reported in Figure S4a. The area of interest was selected with the 'rectangle' tool (Figure S4b) and the RGB counts were measured with the 'Measure' function (Analyze>Measure or Ctrl+M). This procedure opens the 'Results' window containing the maximum, minimum and mean values of for each of the three channels (Figure S4c). The rgb coordinates were calculated from the mean values as described in eq. 3.

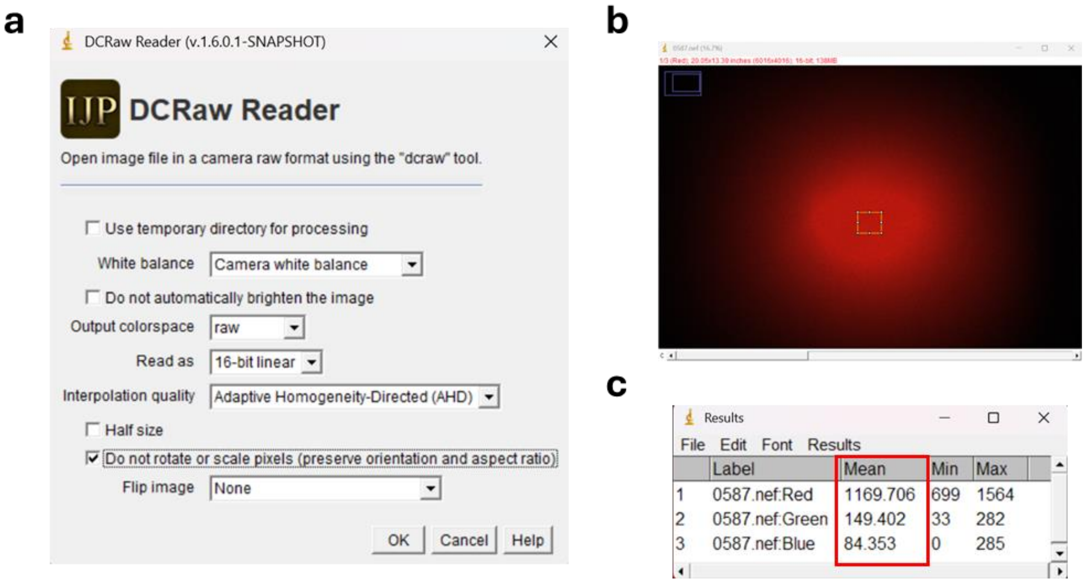

Figure S4. Left: Settings used for image processing in ImageJ. Top-right: Example of the selection of the image's area from which RGB values are extracted. Bottom-right: 'Results' window with the minimum, maximum and mean (highlighted in red) values for the three coordinates.

Spectroscopy: data processing

1) Determination of the camera pseudo matching functions (PCMF): PCMFs were extracted by following a literature method.[5] Monochromatic light was generated using the excitation channel of a spectrofluorimeter with the monochromator slit width set to 1 nm. The monochromator was varied between 380 nm and 720 nm in 10 nm steps. At each step, a spectrum was

recorded using an Optosky ATP2000 CCD spectrometer, and a corresponding photograph was taken with a Nikon D3500 camera by positioning both instruments at the end of the optical path. From each photograph, the RAW RGB channel values were extracted as described above (Figure S5b) and normalized by dividing them by the maximum intensity of the corresponding measured spectrum (Figure S5a). This process yielded three spectral responsivity functions ($\tilde{R}$, $\tilde{G}$, $\tilde{B}$), one for each RGB channel (Figure S5c). These functions were then used to convert spectral data obtained from the Optosky ATP2000 spectrometer into RGB values.

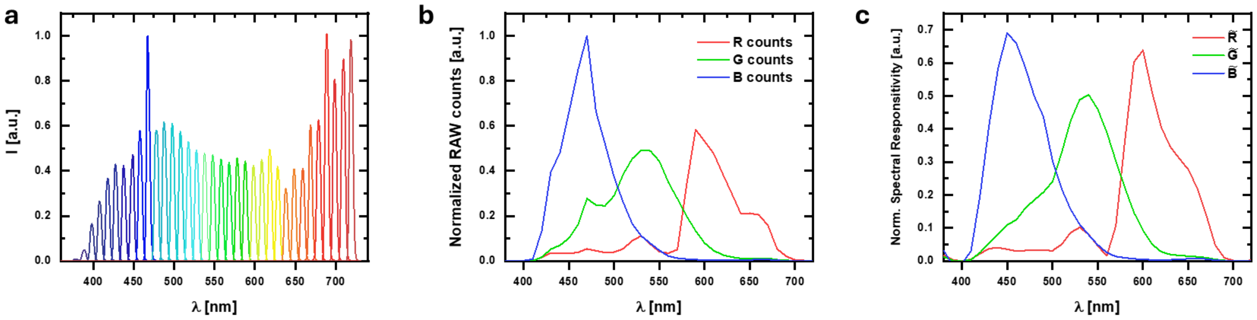

Figure S5. (**a**, **b**) Monochromatic light spectra and normalized plots of RAW counts used for the calculation of the PMFs of the Nikon D3500 camera. (**c**) Calculated PCMFs of the Nikon D3500 camera.

2) Calculation of RGB values from spectra: to calculate the RGB and rgb coordinates from the emission spectrum, the emission spectrum of the fluorophore $I(\lambda)$ is multiplied for each of the three PCMFs ($\tilde{R}$, $\tilde{G}$, $\tilde{B}$) separately. The three resulting functions were integrated between 380 and 720 nm, yielding the RGB coordinates in the camera colour space:

$$R = \int_{380}^{720} I(\lambda)\tilde{R}(\lambda)d\lambda \quad \text{eq. S1}$$

$$G = \int_{380}^{720} I(\lambda)\tilde{G}(\lambda)d\lambda \quad \text{eq. S2}$$

$$B = \int_{380}^{720} I(\lambda)\tilde{B}(\lambda)d\lambda \quad \text{eq. S3}$$

Notice that the calculated RGB values are not normalized and could in principle exceed the value of 255. The chromaticity coordinates are then obtained applying eq. 3.

3) Method validation: to validate the method, we started by measuring the dependence of the rgb coordinates extracted by the photographs from various parameters of the camera. The dependence from the ISO value and the shutter speed is shown in Figure S6. We chose an ISO value of 100-200 and a shutter speed between 0.1 s and 0.025 s.

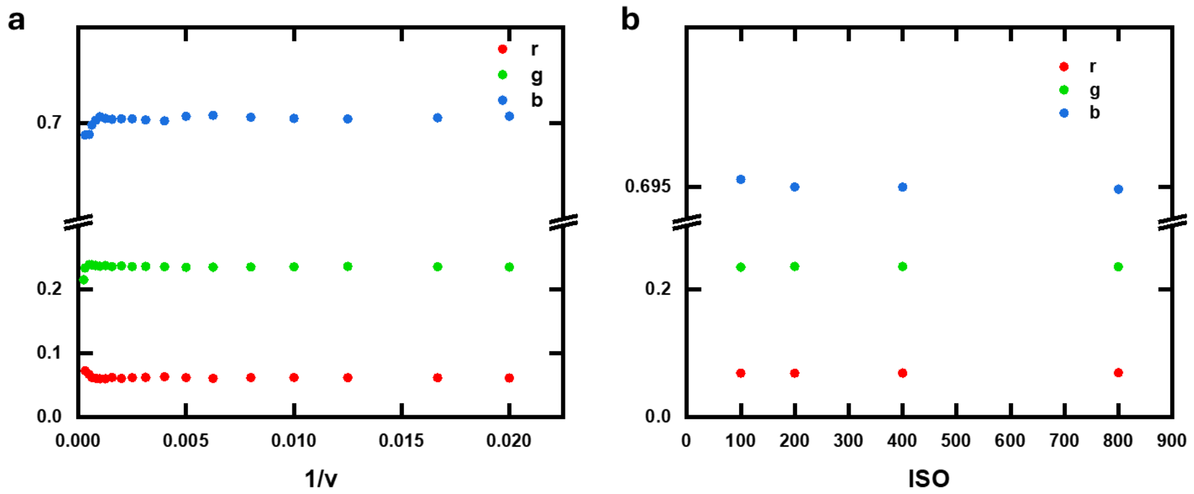

Figure S6. rgb coordinates ratio dependence from the shutter speed (left) and the ISO value (right).

We then extracted the chromaticity coordinates from the emission spectra and the photographs of five different optically inactive chromophores with emission in different parts of the visible region (see spectra in Figure S16) and compare them

to the chromaticity values extracted directly from the photographs. The results are reported in the rg-plane depicted in Figure S7. The data show good correspondence between the two sets, with low standard deviations. We then measured the chromaticity coordinates for the two polarizations of the emission of CsEu(*(−)*-hfbc)$_4$. As for the other chromophores, the spectral and photoscopic data show good accordance. As expected, a significant distance in the rg-plane was observed in this case for L and R polarizations.

Table S2. r/g coordinates for the chromophores used to validate the method

	spectra		photographs	
**Chrom.**	**r (SD)**	**g (SD)**	**r (SD)**	**g (SD)**
**Perylene**	0.06 (0.02)	0.227 (0.003)	0.0595 (0.0001)	0.23581 (0.00001)
**Coumarin 153**	0.326 (0.002)	0.518 (0.007)	0.3300 (0.0004)	0.5191 (0.0003)
**Eu(TTA)$_3$Phen**	0.90 (0.02)	0.090 (0.002)	0.9052 (0.0002)	0.0869 (0.0001)
**Rhodamine B**	0.500 (0.001)	0.444 (0.003)	0.494 (0.009)	0.436 (0.006)
**Tb(PMIP)$_3$**	0.223 (0.003)	0.56 (0.02)	0.228 (0.003)	0.560 (0.007)
**CsEu(*(−)*-hfbc)$_4$ (R-pol)**	0.864 (0.002)	0.107 (0.002)	0.863 (0.002)	0.108 (0.004)
**CsEu(*(−)*-hfbc)$_4$ (L-pol)**	0.857 (0.004)	0.117 (0.004)	0.856 (0.005)	0.119 (0.005)

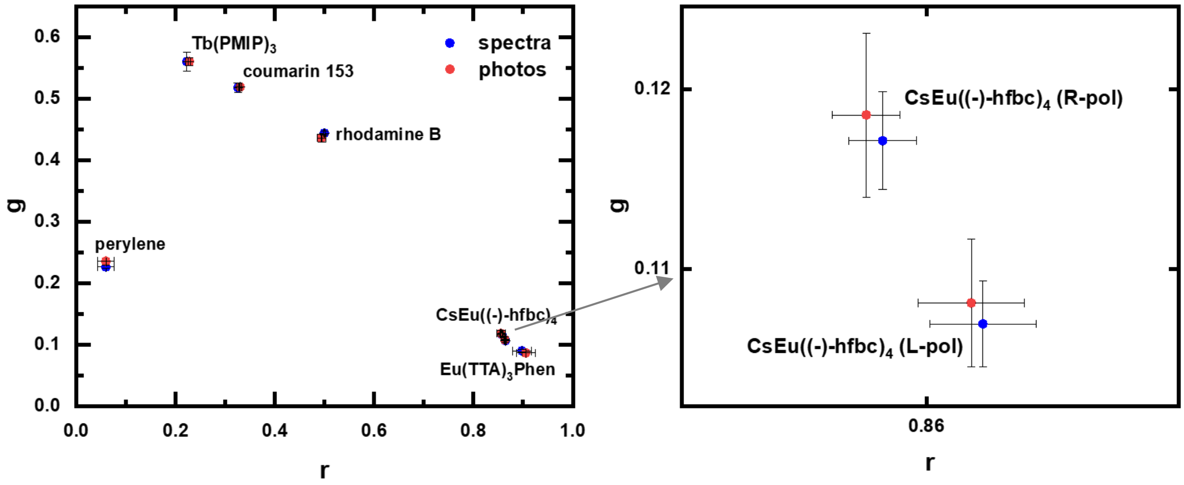

Figure S7. Left: two-dimensional rg-plot for the five chromophores used for the validation of the method, blue points are used to represent coordinates obtained from spectroscopic measurements and red points are used to represent coordinates obtained from photoscopic measurements. Right: Zoomed-in section showing the points for CsEu(hfbc)$_4$ complexes.

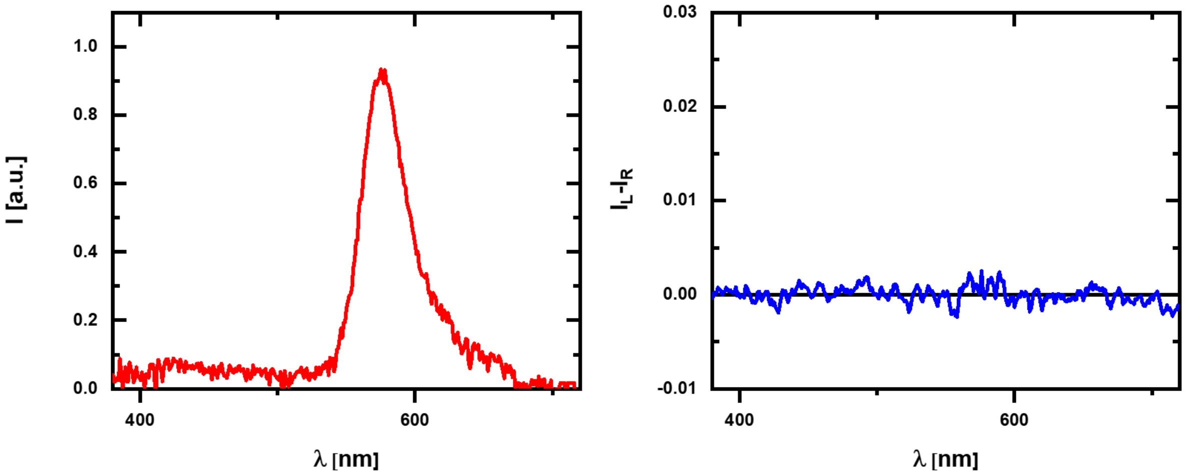

Figure S8. Emission (left) and CPL (right) spectra of a rhodamine B/γ-cyclodextrin aqueous solution under 365 nm excitation.

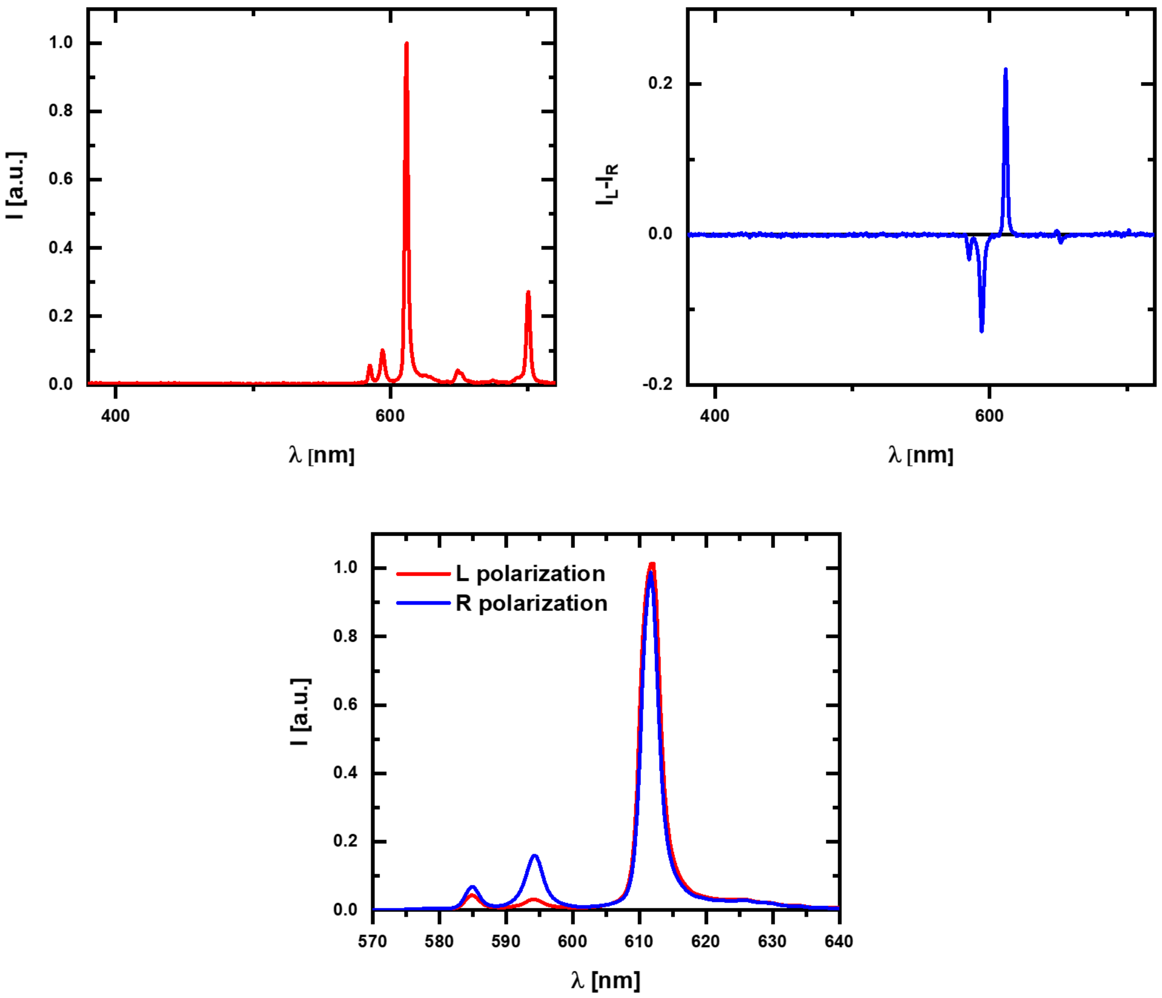

Figure S9. Total emission (top-left), CPL (top-right) and emission under left/right polarization (bottom) for CsEu(*(−)*-hfbc)$_4$ in DCM under 365 nm excitation.

d_{rgb} for perylene/ Eu(TTA)$_3$Phen solution

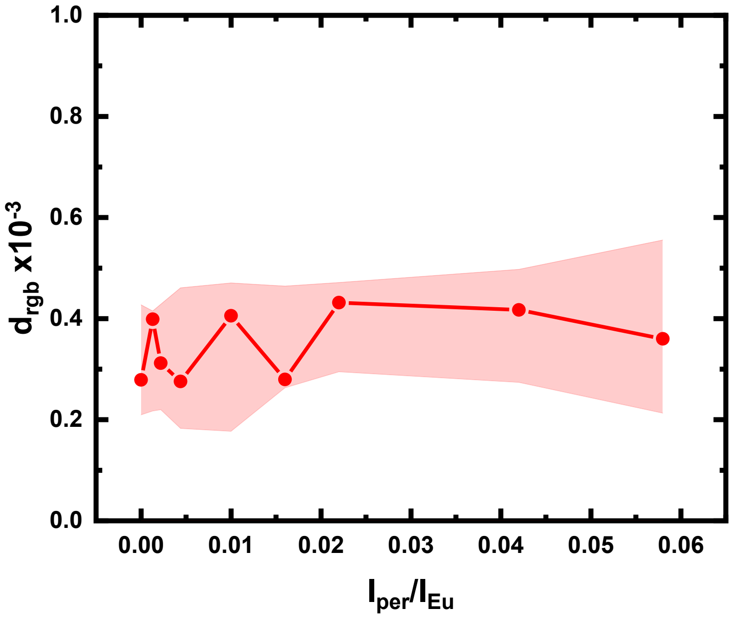

Figure S10. d_{rgb} values for a solution of optically inactive Eu(TTA)$_3$Phen with increasing amounts of perylene.

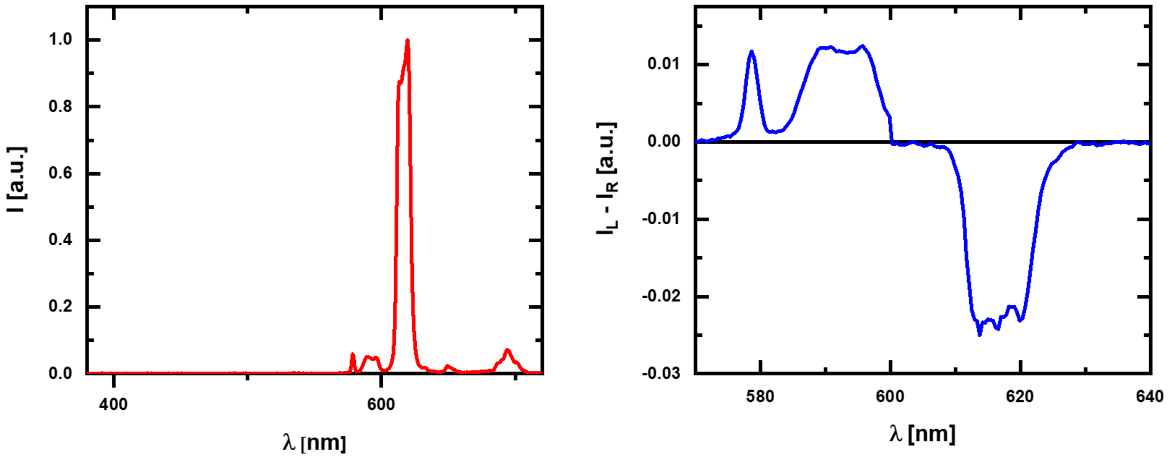

Figure S11. Emission (left) and CPL (right) spectra of Eu(TTA)$_3$(*S,S*)-^{i}PrPyBox in DCM under 365 nm excitation.

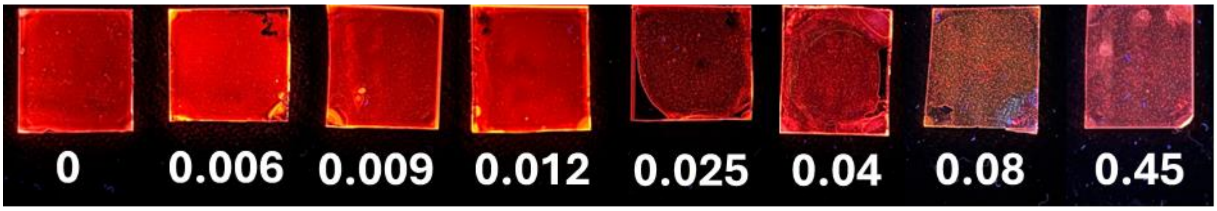

Figure S12. Photographs of Eu(TTA)$_3$(*S,S*)-^{i}PrPyBox/perylene films for different I[Perylene]/I[Eu(TTA)$_3$(*S,S*)-^{i}PrPyBox] ratio.

d_{rgb} data from spectroscopic measurements

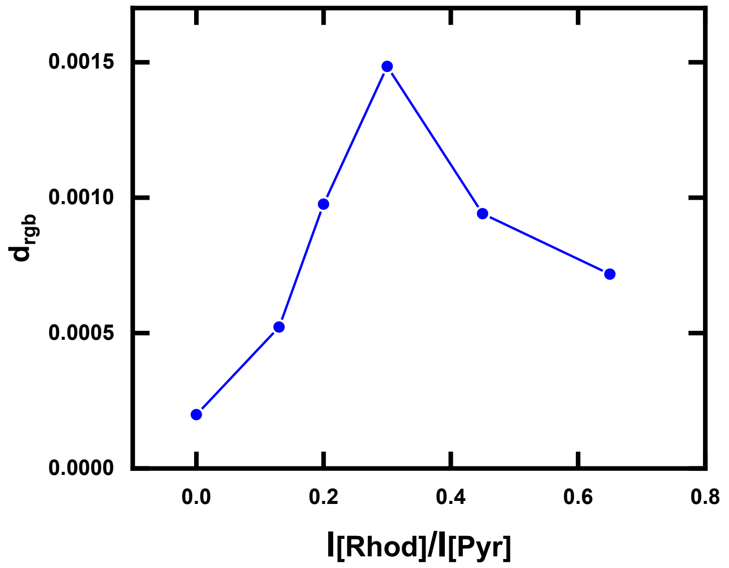

Figure S13. d_{rgb} spectral data for a solution of rhodamine B and pyrene/γCDx, to be compared with photoscopy results in Figure 2a.

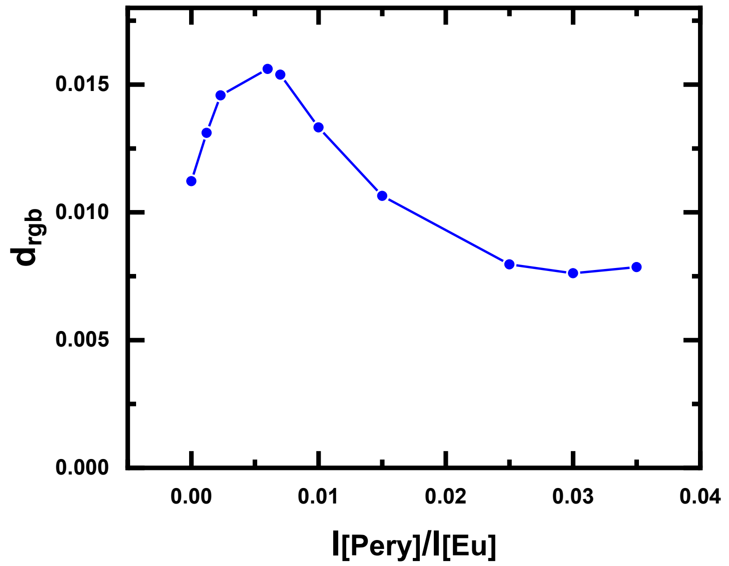

Figure S14. d_{rgb} spectral data for a solution of perylene and CsEu(*(−)*-hfbc)$_4$, to be compared with photoscopy results in Figure 3a.

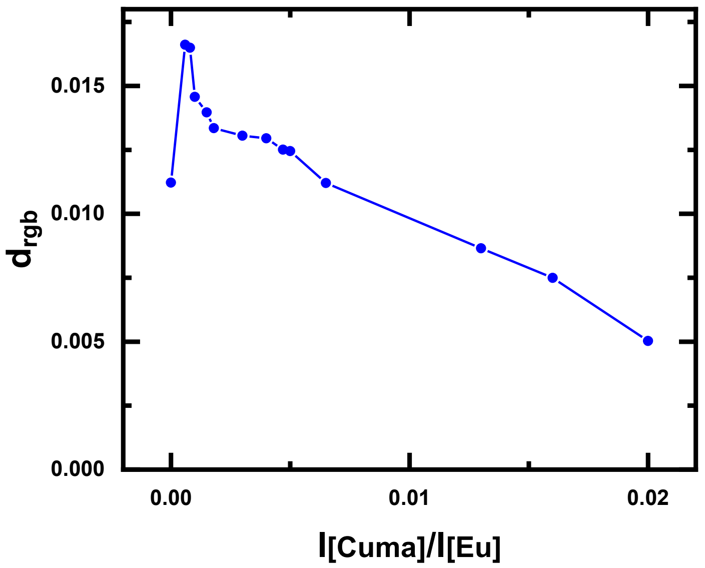

Figure S15. d_{rgb} spectral data for a solution of coumarin 153 and CsEu(*(−)*-hfbc)$_4$, to be compared with photoscopy results in Figure 4a.

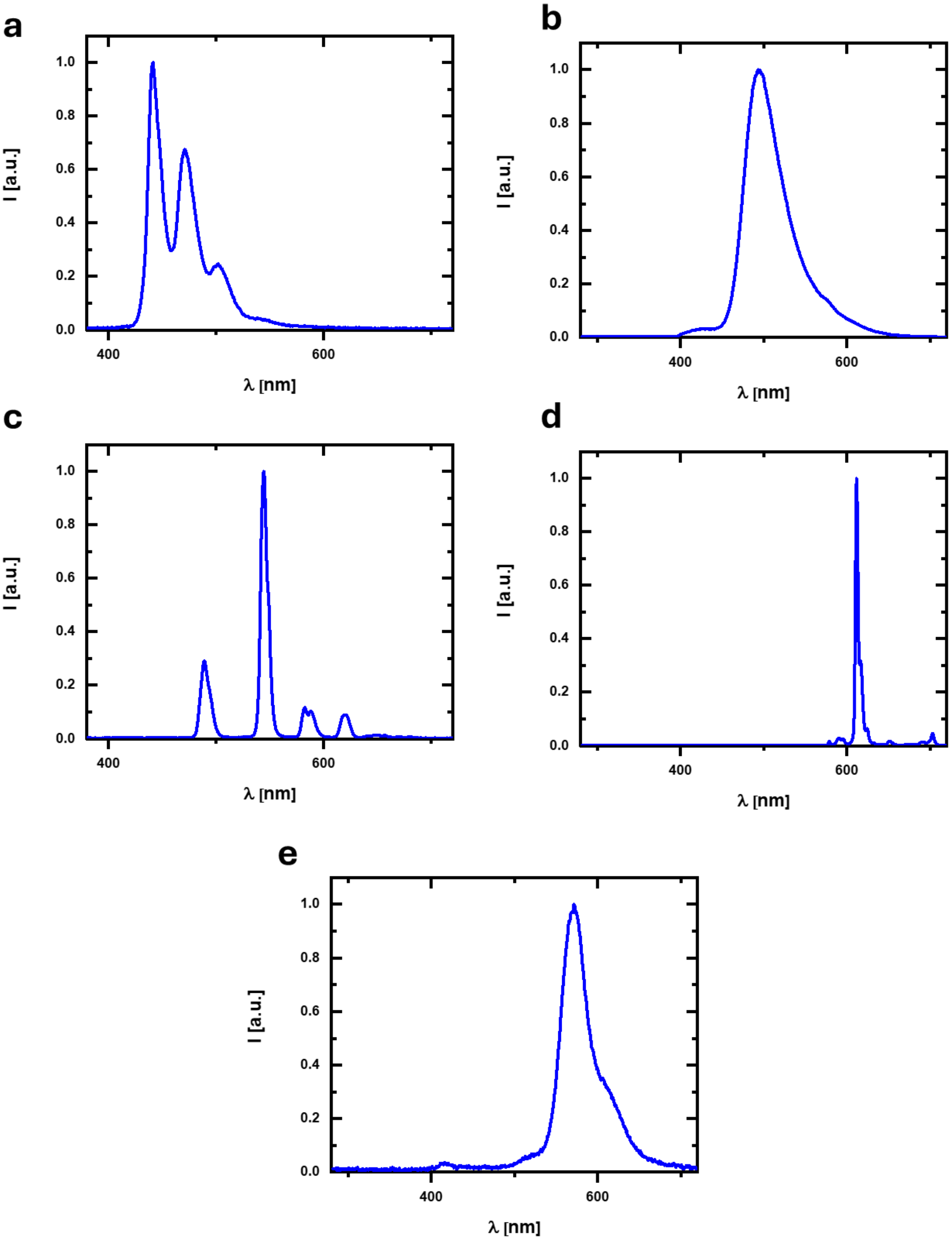

Figure S16. Emission spectra of (**a**) perylene in DCM, (**b**) coumarin 153 in DCM, (**c**) Tb(PMIP)$_3$ in MeOH, (**d**) Eu(TTA)$_3$Phen in DCM and (**e**) rhodamine B in DCM under 365 nm excitation.

d_{rgb} dependence on the distance between the polarized and non-polarized bands

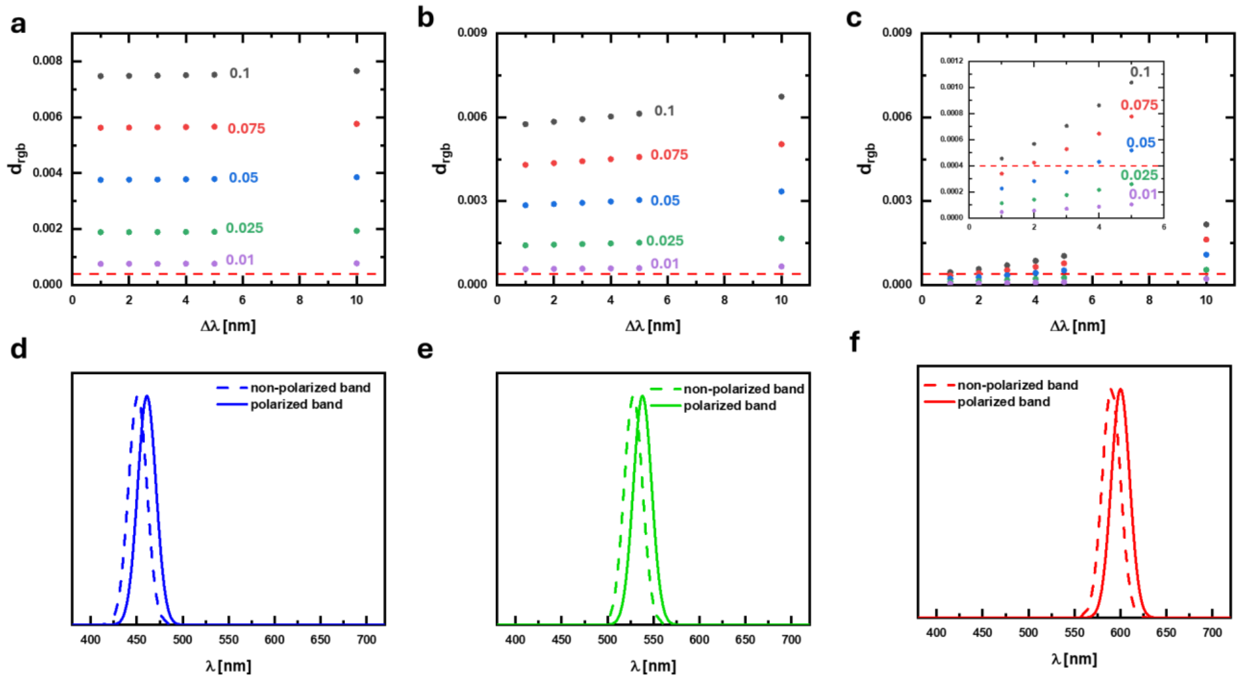

Figure S17. (**a**-**c**) Simulated plots of d_{rgb} against the spectral distance between the maximum of the polarized and non-polarized band (Δλ) for various g_{lum} values. The horizontal dashed red lines show the limit of detection for d_{rgb}. (**d**-**f**) Spectra displaying the polarized band (solid line) and the non-polarized band (dashed line). The plots were obtained by varying the wavelength of the non-polarized band with the polarized band centered at the maximum of the three spectral responsivity functions of the camera (**a** blue, **b** green, **c** red).

d_{rgb} dependence on the deviation from the alignment angle between LP and QWP

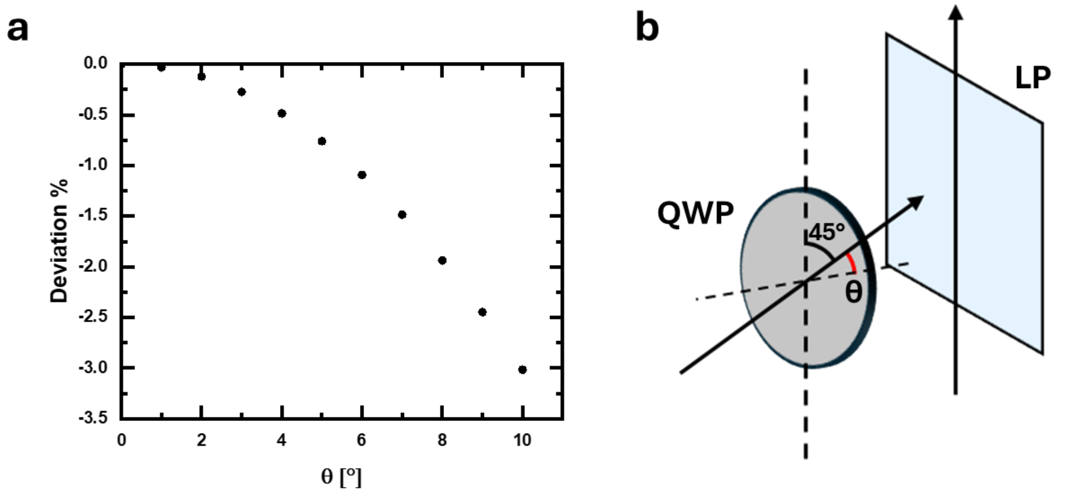

Figure S18. (**a**) Deviation from the true value of d_{rgb} (as a percentage of the real value of d_{rgb}) plotted against the distance from the right angle of alignment between the LP and QWP axis. (**b**) Schematic depiction of the setup, θ is highlighted in red.

d_{rgb} dependence from $\tilde{g}_{lum}$

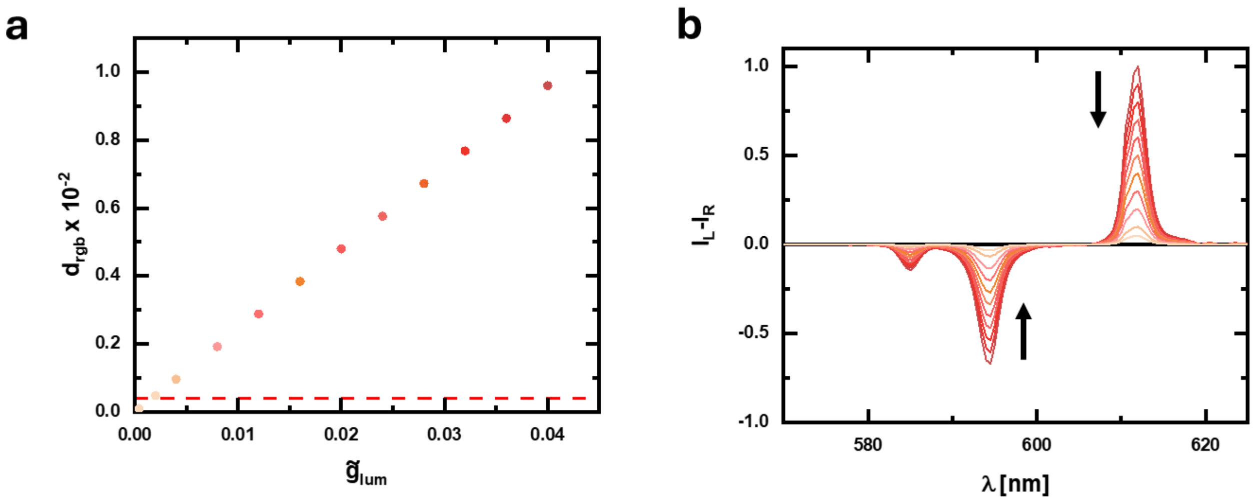

Figure S19. (**a**) d_{rgb} plotted against $\tilde{g}_{lum}$ the horizontal dashed red lines show the limit of detection for d_{rgb}. (**b**) Corresponding CPL spectra.

Extraction of single band glum from photoscopy data

As an example, we took the example in Figure 2a (Pyr/CDx + Rhodamine B). Considering the total emission spectrum corresponding to [rhodamine B]/[pyrene] emission intensity ratio of ≈0.3 (maximum d_{rgb}), we prepared a calibration curve by computing the expected d_{rgb} for several g_{lum} associated to the pyrene excimer band (Figure 2b), then we extracted the g_{lum} corresponding to an observed d_{rgb}=1.4·10^{-3}. In this way, we found a g_{lum} of 0.022 to be compared with the g_{lum}= 0.025 observed through regular CPL measurements.

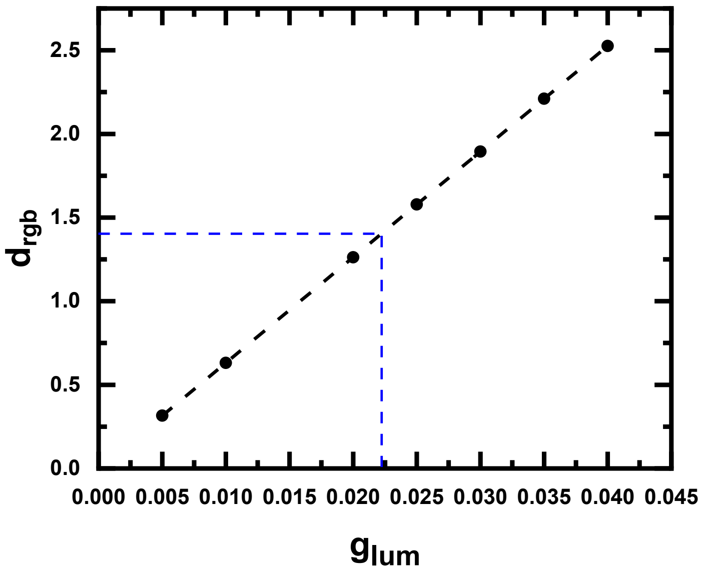

Figure S20. Calibration curve obtained by computing the expected d_{rgb} for pyrene different g_{lum} in the Pyr/CDx + Rhodamine B mixture. The blue dashed lines show the extrapolation of the g_{lum} associated with the measured d_{rgb} value.

References

(1) Willis, O. G.; Pucci, A.; Cavalli, E.; Zinna, F.; Di Bari, L. Intense 1400–1600 nm Circularly Polarised Luminescence from Homo- and Heteroleptic Chiral Erbium Complexes. *J. Mater. Chem. C* **2023**, *11* (16), 5290–5296.

(2) Müller, P.; Boléa, C. Carbenoid Pathways in Copper-Catalyzed Intramolecular Cyclopropanations of Phenyliodonium Ylides. *Helv. Chim. Acta* **2001**, *84* (5), 1093–1111.

(3) Zinna, F.; Giovanella, U.; Di Bari, L. Highly Circularly Polarized Electroluminescence from a Chiral Europium Complex. *Adv. Mater.* **2015**, *27* (10), 1791–1795.

(4) Ye, Z.; Chen, J.; Wang, G.; Yuan, J. Development of a Terbium Complex-Based Luminescent Probe for Imaging Endogenous Hydrogen Peroxide Generation in Plant Tissues. *Anal. Chem.* **2011**, *83* (11), 4163–4169.

(5) Schwaebel, T.; Menning, S.; Bunz, U. H. F. Photoscopy: Spectroscopic Information from Camera Snapshots? *Chem. Sci.* **2014**, *5* (4), 1422–1428.
